# Viral Infection of Human Natural Killer Cells

**DOI:** 10.3390/v11030243

**Published:** 2019-03-12

**Authors:** Elisabeth A. van Erp, Mirjam R. van Kampen, Puck B. van Kasteren, Jelle de Wit

**Affiliations:** 1 Centre for Infectious Disease Control, National Institute for Public Health and the Environment (RIVM), 3721 MA Bilthoven, The Netherlands; liz.van.erp@rivm.nl (E.A.v.E.); mirrvk@gmail.com (M.R.v.K.); puck.van.kasteren@rivm.nl (P.B.v.K.); 2 Section Pediatric Infectious Diseases, Laboratory of Medical Immunology, Radboud Institute for Molecular Life Sciences, Radboudumc, 6525 GA Nijmegen, The Netherlands; 3 Radboud Center for Infectious Diseases, Radboudumc, 6525 GA Nijmegen, The Netherlands

**Keywords:** NK cells, virus, infection, immune evasion, receptors, effector functions

## Abstract

Natural killer (NK) cells are essential in the early immune response against viral infections, in particular through clearance of virus-infected cells. In return, viruses have evolved multiple mechanisms to evade NK cell-mediated viral clearance. Several unrelated viruses, including influenza virus, respiratory syncytial virus, and human immunodeficiency virus, can directly interfere with NK cell functioning through infection of these cells. Viral infection can lead to immune suppression, either by downregulation of the cytotoxic function or by triggering apoptosis, leading to depletion of NK cells. In contrast, some viruses induce proliferation or changes in the morphology of NK cells. In this review article, we provide a comprehensive overview of the viruses that have been reported to infect NK cells, we discuss their mechanisms of entry, and describe the interference with NK cell effector function and phenotype. Finally, we discuss the contribution of virus-infected NK cells to viral load. The development of specific therapeutics, such as viral entry inhibitors, could benefit from an enhanced understanding of viral infection of NK cells, opening up possibilities for the prevention of NK cell infection.

## 1. Introduction

Natural killer (NK) cells are innate lymphocytes that represent the first line of defense against tumor cells and viral infections [1,2]. The importance of NK cells in the antiviral immune response is underscored by the increased susceptibility to viral diseases of patients with a congenital NK cell deficiency. Although NK cell deficiencies are rare, multiple cases have been described in which increased susceptibility to numerous herpesviruses is shown, which has been extensively reviewed elsewhere [3].

NK cells have multiple mechanisms to kill virus-infected cells, including the engagement of extracellular death receptors and exocytosis of cytolytic granules [4]. To mediate cytolysis through engagement of death receptors expressed on target cells, NK cells express multiple extracellular ligands, including Fas ligand (FasL) and the tumor necrosis factor-related apoptosis-inducing ligand (TRAIL) [5]. Viral infection, for example by cytomegalovirus (CMV) and encephalomyocarditis virus (EMCV) [4], can induce the expression of death receptors on infected cells, which can subsequently interact with FasL and TRAIL on NK cells, resulting in apoptosis of the target cell. The other route to induce cytotoxicity is through the release of stored cytolytic granules that contain perforin and granzymes that enter the target cell and trigger apoptosis through caspase-mediated signaling pathways [4]. In addition to cytotoxicity, NK cells contribute to the antiviral response through the release of a wide range of proinflammatory cytokines with antiviral activity [6].

Activation of NK cells is regulated by a balance in the engagement of its activating and inhibitory receptors in combination with the presence of certain cytokines. Together, these stimuli determine the type and strength of NK cell activity [7].

Healthy cells inhibit NK cell activation mainly through the expression of major histocompatibility complex class I (MHC-I) molecules, which interact with inhibitory receptors present on the NK cell surface. Inhibitory NK cell receptors that ligate to MHC-I include killer cell immunoglobulin-like receptors (KIRs) and leukocyte immunoglobulin-like receptors (LILRs) [7]. This inhibitory receptor-mediated signaling is essential to counteract activating signaling in order to protect against NK cell over-activity. Some viruses are known to downregulate surface expression of MHC-I to interfere with the presentation of viral antigens, thereby escaping detection by the adaptive immune system [8]. Although this immune evasion strategy is effective in preventing recognition by T cells, decreased MHC-I expression promotes the recognition and clearance of virus-infected target cells by NK cells [9]. The concept of target cell recognition via the absence of inhibitory MHC-I engagement is known as the “missing-self” hypothesis.

The activating receptors that are expressed by NK cells facilitate activation upon detection of viral or stress-induced ligands on target cells. For example, the natural cytotoxicity receptors (NCRs), including NKp46, NKp44, and NKp30, are known to bind viral glycoproteins [10,11], allowing for activation of NK cells upon detection of infected cells. In addition, NK cells are activated through binding to antibody-opsonized target cells with Fc-γ receptor IIIA (FcγRIIIA), which induces antibody-dependent cell-mediated cytotoxicity (ADCC). 

Due to the important role of NK cells in the early antiviral immune response, viruses have evolved numerous strategies to evade NK cell effector functions. One of these evasion strategies is the manipulation of NK cells through direct infection. In this review, we provide a comprehensive overview of the viruses that have been reported to infect NK cells. We discuss their mechanisms of entry, describe how they affect NK cell function, and indicate which viruses deplete NK cells through the induction of apoptosis. Moreover, we address the contribution of infected NK cells to viral load.

## 2. Entry Mechanisms

Viruses have evolved many mechanisms to enter host cells. The best-known mechanism is entry through binding to specific receptors, which either leads to fusion directly at the plasma membrane, or to entry following clathrin- or caveolin-dependent endocytosis of the viral particle. Additionally, viruses may require direct cell–cell interactions for infection. Finally, viral entry may occur through macropinocytosis, which is the nonspecific uptake of extracellular material. Hence, non-specific binding of virus can lead to internalization, even in the absence of specific viral entry receptors on the target cell. Macropinocytosis has been described for multiple viruses [12], but to date this has not been identified as a strategy to enter NK cells. The different entry mechanisms that have been shown for viral infection of NK cells are described below and summarized in Table 1.

### 2.1. Receptor-Mediated Viral Entry

One of the entry pathways for viruses is through binding of cellular receptors by viral proteins, which leads to receptor-mediated entry. NK cells express multiple receptors that are known to be used for virus entry. Additionally, it has been described that NK cells acquire entry receptors from infected cells, either through direct contact via the immunological synapse or by exosome transfer (extensively reviewed in Reference [13]).

Human influenza A virus (IAV) is an enveloped virus that has been shown to directly infect both human and mouse NK cells in vitro [14,15]. In vivo infection of NK cells was demonstrated in several mouse studies [15,16]. Viral entry by IAV into NK cells from human peripheral blood mononuclear cells (PBMCs) was found to be mediated by clathrin- and caveolin-dependent endocytosis [14]. IAV virions generally infect respiratory tract epithelial cells by binding of viral hemagglutinin (HA) protein to α-2,3 and/or α-2,6 linked terminal sialic acids, which are found in abundance on the surface of respiratory tract epithelial cells [17]. It has been confirmed that both forms of sialic acids are also present on the surface of NK cells [15]. In addition, the α-2,6 terminal sialic acids are present on activating NK cell receptor NKp46 [11]. Indeed, free HA binding and internalization into NK cells was shown to be terminated by sialidase treatment, demonstrating dependence on the sialic acid interaction [18]. Interestingly, the interaction of NKp46 with HA on the surface of virus-infected cells is a known mechanism for NK cell recognition and killing of target cells [11], but it appears that the IAV virion itself is able to infect NK cells by internalization of the virion through the same interaction with NKp46 [18].

Human immunodeficiency virus 1 (HIV-1) has also been shown to infect human NK cells both in vitro [19] and in vivo [20]. HIV-1 is an enveloped virus that enters cells by binding with its viral envelope glycoprotein gp120 to the CD4 receptor, and to one of the co-receptors C-X-C chemokine receptor 4 (CXCR4) or C-C chemokine receptor 5 (CCR5) [20]. Virus binding to CD4 brings the virion within range of the co-receptors which then facilitate membrane fusion and subsequent release of the viral core into the host cell cytoplasm. Due to abundant expression of co-receptor CXCR4, NK cells are mostly susceptible to CXCR4-tropic strains of HIV-1 [21]. However, CCR5-tropic strains were also found to infect NK cells, after upregulation of CCR5 expression on NK cells [21,22]. CCR5 expression was either induced by stimulation of NK cells with feeder cells in presence of IL-2 [22], or by depletion of C-C chemokine ligands (CCL) that inhibit CCR5 expression [21]. The CD4 receptor is not commonly expressed within the NK lineage, but infection with human herpesvirus 6 (HHV-6, family Herpesviridae) can upregulate CD4 expression in NK cells [23]. It is thought that this HHV-6 infection primes NK cells for HIV infection [23]. Alternatively, a CD4-independent pathway may facilitate HIV-1 entry in absence of CD4 [19]. 

Epstein Barr virus (EBV, family Herpesviridae) is a lymphotropic virus that is present in a variety of lymphocyte neoplasms including NK cell lymphoma [24], indicating that EBV can infect NK cells in vivo. Moreover, latently infected non-neoplastic NK cells have been detected in patients with primary EBV infection [25]. The entry mechanism for NK cells is under debate as EBV usually infects oropharyngeal epithelial cells and B cells. The EBV envelope glycoprotein gp350/220 interacts with the CD21 receptor on the surface of B cells and subsequent interaction of the viral gp85-gp25-gp42 glycoprotein complex with MHC-II molecules triggers membrane fusion (reviewed in [26]). Whereas NK cells do express MHC-II molecules [27,28], they do not express CD21 on their cell surface. Interestingly, one study indicated the acquisition of the CD21 receptor by NK cells upon interaction with CD21-positive, EBV-infected cells [29]. Others show a CD21-independent mechanism of NK cell infection by EBV [30].

The receptor-mediated entry of IAV, HIV, and EBV into NK cells indicates that NK cells are permissive for infection due to expression or acquisition of virus entry receptors. However, for many other viruses known to infect NK cells, no receptor has been identified yet. Our group recently showed that RSV can also infect NK cells in vitro and that infection is enhanced in the presence of sub-neutralizing concentrations of RSV-specific antibodies [42]. This study proposes that FcγRIIIA, the IgG-receptor expressed on NK cells, mediates binding to NK cells in the presence of virus-specific antibodies [42]. Although to date it has not been described for other viruses, antibody-mediated entry could be exploited by many more viruses as a mechanism to enter NK cells in individuals that carry virus-specific antibodies. 

### 2.2. Cell–Cell Interaction-Mediated Viral Entry

NK cells closely interact with infected cells, for example through the interaction between death receptors and their ligands, in order to clear the viral infection. During this interaction, they may acquire membrane fragments that contain entry receptors for viruses. This transfer of entry receptors between cells could take place via the immunological synapse, or via intercellular transfer of exosomes (reviewed in Reference [13]). In addition, cell–cell interaction may facilitate direct virus entry through the immunological synapse. 

The human T cell leukemia virus 1 (HTLV-1, family Retroviridae) is known to require cell–cell interactions for infection, as infected cells produce virtually no cell-free infectious viral particles [47]. In an extensive imaging study by Igakura and colleagues, the HTLV-1 core complexes and genome were shown to accumulate at cell–cell junctions and were subsequently transferred to uninfected cells [48]. This transfer mechanism was not only observed between T cells, the main target for HTLV, but also between CD4^+^ T cells and NK cells [48]. Earlier studies had also shown transfer of HTLV-1 from T cells to NK cells, but only after activation of the NK cells with an anti-FcγRIII antibody [49].

Vaccinia virus (VV, family Poxviridae) was also proposed to spread to NK cells through cell–cell contact [43,44]. This virus is able to enter various cell types through non-specific macropinocytosis [50]. VV has been shown to infect NK cells in vitro, but only after co-culturing with other, infected, cell types [43,44]. It was suggested that NK cells become infected by VV upon interaction with infected target cells [43]. These observations imply that there is a transfer of VV particles through the immunological synapse or via exosomes. It must be noted, however, that acquisition of virus entry receptors by NK cells through cell–cell interactions cannot be ruled out for VV. 

Two other cell-associated viruses are herpes simplex virus (HSV, family Herpesviridae) and varicella zoster virus (VZV, family Herpesviridae). HSV was shown to enter NK cells after co-culture with infected fibroblasts [36]. Incubation of NK cells with cell-free HSV only induced NK cell lysis at MOIs >100. VZV infection of NK cells in vitro was recently shown after co-culture with infected epithelial cells [37]. Additionally, cell-free direct infection was observed in the case of VZV, indicating that multiple entry mechanisms may be involved. 

Although some of these viruses are known to produce virtually no cell-free infectious viral particles, an indirect effect of other infected cell types cannot be ruled out. Infected cells may secrete multiple soluble mediators (e.g., cytokines and chemokines) that could prime NK cells and make them more prone to infection. Significantly higher NK cell infection rates are seen after stimulation with IL-2 for VZV, and after stimulation with IL-15 for RSV, compared to unstimulated cells [37,42] [unpublished observations]. In conclusion, additional experiments are needed to establish whether infection through co-culture of infected cells with NK cells is a direct or indirect effect.

### 2.3. Unknown Internalization Mechanism

There are many more viruses found to infect NK cells, but research into their entry mechanisms is lacking. DNA of the asymptomatic Torque teno virus (TTV, family Anelloviridae), also known as the transfusion-transmitted virus, has been found in NK cells of viremic individuals [31]. RNA from human pegivirus (HPgV), a virus of the Flaviviridae family formerly known as hepatitis G virus, was detected in NK cells from infected individuals [32]. Furthermore, vesicular stomatitis virus (VSV, family Rhabdoviridae) [45], measles virus (MV, family Paramyxoviridae) [40], human cytomegalovirus (CMV, family Herpesviridae) [33], and HHV-6 [23] have been reported to infect NK cells in vitro, but their entry mechanisms are unknown. Entry in the absence of a virus entry receptor may be explained by macropinocytosis, as no specific entry receptor is required for this process [12]. Respiratory syncytial virus (RSV) has been shown to enter epithelial cells through macropinocytosis [41]. Whether entry of NK cells by RSV is mediated through macropinocytosis has not been investigated. In summary, these studies indicate that viral infection of NK cells is not a rare phenomenon but widespread across various virus families. 

## 3. Modulation of NK Cell Function and Phenotype

Because of the prominent role of NK cells in antiviral immunity, viruses have evolved multiple evasion strategies that affect NK cell effector functions and phenotype. Both these aspects are addressed below and are summarized in Table 1.

### 3.1. Influence on Effector Function

Inhibition of cytotoxicity is the most prominent effect caused by viral infection of NK cells. In vitro infection by IAV results in decreased cytotoxic function and induction of apoptosis in both adult and cord blood NK cells [14,15,38]. A follow-up study demonstrated that interaction with either the intact influenza virion or free HA protein inhibits NK cell cytotoxicity in a dose-dependent manner through downregulation of the NKp46 and NKp30 signaling pathway [18]. Besides human IAV, avian IAV virions or free avian HA protein also inhibit human NK cell cytotoxicity [39]. Moreover, infection with avian IAV induces apoptosis of NK cells [39]. Thus, these studies demonstrate that binding of IAV is enough to inhibit cytotoxicity, whereas NK cell infection leads to depletion of NK cells through apoptosis. This cellular depletion is supported by evidence of decreased NK cell numbers in both the peripheral blood and lungs of IAV-infected patients [51,52].

Similar effects were demonstrated for VV infection, which also leads to decreased cytotoxicity [43]. However, incubation with UV-inactivated VV did not affect cytotoxicity, indicating that VV replication is required for inhibition [43]. This decreased cytotoxicity was a result of increased NK cell sensitivity to inhibitory killer immunoglobulin-like receptor (KIR) signaling, induced by active viral replication. In addition, decreased perforin secretion was shown for RSV-infected adult NK cells compared to uninfected NK cells [42]. The use of a fusion inhibitor in this study indicated that the mere binding or presence of the virus was not enough to inhibit perforin secretion.

Decreased ability to lyse target cells has also been reported for HSV and MV-infected NK cells [36,40]. The authors did not investigate the effect of non-infectious viral particles, therefore it is unclear whether infection of NK cells was required to inhibit cytotoxicity. For HIV, only a limited effect on the cytotoxic response in vitro was observed in two independent studies [19,21]. The authors propose that the modest impact on killing capacity in vitro was due to the low infection levels that were established [19,21], as depressed NK cell cytotoxicity has been detected in patients with AIDS [53,54,55,56]. In addition, viruses can affect NK cell function indirectly by inducing either proliferation or apoptosis. HTLV and HPgV induce increased proliferation and prolonged survival [49,57], whereas in many other in vitro infections apoptosis is induced, as seen for HIV [19], HHV-6 [23], and IAV [14]. Depletion of NK cells during viral infections has been shown in clinical studies for IAV [51,52] and RSV [51,58], but whether this is due to direct infection of NK cells is unknown.

Besides their cytotoxic potential, NK cells are also prominent cytokine and chemokine secreters. Multiple viruses have been shown to affect cytokine secretion. In the presence of IAV, a downregulation was observed for the production of multiple proinflammatory cytokines and chemokines including IFN-γ, GM-CSF, MIP-1α (CCL3), MIP-1β (CCL4), and RANTES (CCL5) [15]. On the contrary, another study showed an increase in IFN-γ and TNF-α in the cell supernatant after in vitro IAV infection, although this increase was mainly dependent on the presence of IL-15 [38]. In addition, it must be noted that no distinction was made between IAV-infected and IAV-exposed NK cells, and differences in infection levels and the influenza strain used could potentially explain the difference between these studies.

Besides IAV, there are more viruses that increase or suppress NK cell IFN-γ production. RSV-infected NK cells have an increased IFN-γ production, compared to RSV-exposed uninfected or mock-infected NK cells [42]. The combination with decreased perforin secretion of RSV-infected NK cells suggests that RSV infection of NK cells induces a proinflammatory rather than a cytotoxic response that could contribute to the immunopathology seen in vivo. Conversely, incubation of NK cells with HPgV seems to decrease IFN-γ production, but this effect is independent of infection [57].

Taken together, it seems that viruses have evolved multiple mechanisms to inhibit NK cell cytotoxicity or induce apoptosis, which could serve as an important immune evasion strategy. Additionally, the effect of viruses on effector cytokine production by NK cells can vary per virus, leading to variable polarization of the NK cells and (ultimately) in skewed steering of other immune cells.

### 3.2. Influence on Phenotype

Virus infection does not necessarily modulate NK cell effector functions directly, but often causes changes in receptor expression. As the balance in activating and inhibiting receptor signaling is crucial for NK cell activity, these phenotypical changes may indirectly influence effector functions. Additionally, the phenotype of infected NK cells can be altered by changes in morphology or even transformation into malignant cells.

Downregulation of the expression of activating NK cell receptors has been described for RSV [42]. Independent of infection, the presence of RSV or RSV-antibody complexes results in lower NKG2D and NKp44 expression levels. In addition, upon infection of NK cells, the expression of (inhibitory) KIRs (KIR3DL1 and KIR2DL2/L3/S2) is upregulated [42]. Functional data in this study show upregulated IFN-γ production and decreased perforin secretion, but whether this is a direct consequence of the phenotypical changes has not been investigated [42]. Downregulation of activating NK cell receptor NKp46 has been described after IAV exposure of adult and cord blood NK cells [38]. This NKp46 downregulation was accompanied by increased IFN-γ secretion, decreased perforin expression and decreased cytotoxicity [38]. However, a direct link between phenotype and functionality was not investigated. In contrast to these findings, a study performed by Mao and colleagues did not show altered NKp46 expression on NK cells after exposure to IAV virions [18]. An additional study reported no differences in activating (NCR1, NKG2D, NKRP1c, CD244c and Ly49D) or inhibiting (NKG2A, Ly49A, Ly49C/I, and Ly49G2) receptor expression after IAV infection of NK cells, but NKp46 expression was not investigated [15]. Although no difference in receptor expression was found, the cytotoxic potential of NK cells was downregulated in both these studies [15,18]. Incubation of NK cells with VZV leads to decreased CD56 and FcγRIII expression, in combination with an increased expression of CD57, which is a hallmark of NK cell maturity [37]. In addition, VZV induces the expression of skin-homing chemokine receptors CCR4 and cutaneous lymphocyte antigen (CLA) on infected NK cells, thereby promoting a skin-homing phenotype [37]. Data supporting a functional effect of these phenotypical changes is lacking. Viral infection of NK cells has also been shown to cause de novo expression of receptors that are not intrinsically expressed within the NK lineage. HHV-6 induces CD4 expression in infected NK cells, enabling subsequent (CD4-dependent) HIV infection [23]. The effect of CD4 expression on functionality of the HHV-6-infected NK cells was not investigated. 

Changes to the morphology of NK cells have been reported following EBV infection in vitro, where infection of NK cells caused cell deformation and increased size [30]. Moreover, EBV infection in vivo has been linked to NK cell malignancies [34,35], suggesting the involvement of EBV in transformation of NK cells.

These studies indicate that each virus induces a different phenotype, often skewed towards an NK cell state that promotes the establishment of a successful infection. 

## 4. Contribution to Viral Load

Replication of the viral genome, transcription of viral proteins, and subsequent release of new infectious virions are necessary for virus spread. Since NK cells are infected, they may also contribute to the viral load. A significant role for production of virus particles within NK cells has not been described for the majority of viruses mentioned in this review. Nevertheless, in vitro studies have observed (limited) production of infectious viral particles by NK cells infected with HIV-1 [21,46], VZV [37], and VSV [45]. HIV-1-infected NK cells produce infectious HIV in vitro and depletion of NK cells from PBMC results in significant reduction of HIV-1 propagation [21,46]. VZV-infected NK cells were able to transmit infectious virus to epithelial cells, suggesting productive infection by VZV [37]. Production of infectious VSV particles has only been investigated in NK cell lines, where VSV infection seemed to result in a persistent infection that continued to produce infectious VSV for over a year [45]. Taken together, it is currently unknown whether infected NK cells serve as a reservoir for certain viral infections and contribute significantly to the dissemination of the virus. Although HIV-1, VZV and VSV seem to establish productive infections, it remains to be investigated whether the amount of released infectious viral particles is enough to significantly contribute to the viral load in vivo.

## 5. Conclusion and Future Perspectives

**Figure 1:**
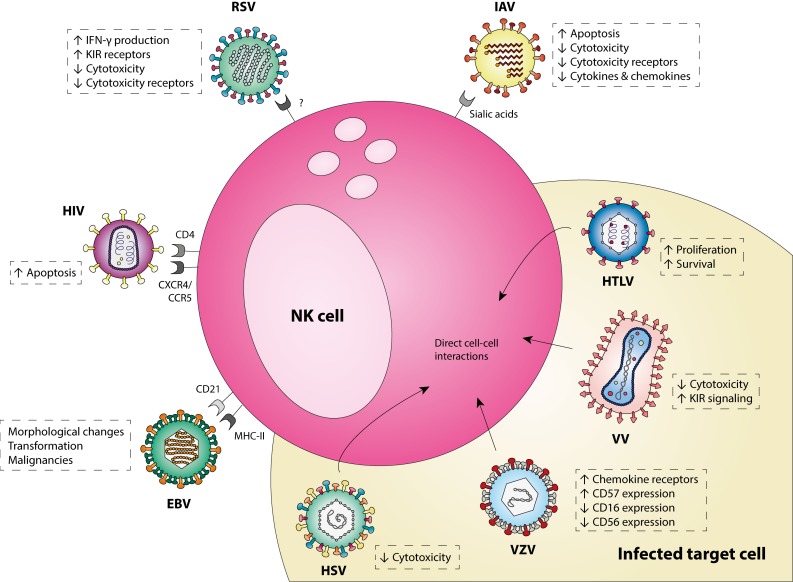
A schematic representation of the most-studied viruses that infect NK cells. EBV, Epstein Barr virus; HIV, human immunodeficiency virus; HSV, herpes simplex virus; HTLV, human T-lymphotropic virus; IAV, influenza A virus; RSV, respiratory syncytial virus; VV, vaccinia virus; VZV, varicella zoster virus.

Accumulating evidence demonstrates that NK cells can serve as target cells for viral infections. Viruses have evolved multiple mechanisms to enter NK cells, change the cellular effector function and affect the subsequent immune response. Literature discussed in this review shows that viruses from nine different families can infect NK cells, indicating this is not an uncommon phenomenon. An overview of the most thoroughly studied viruses that infect NK cells and their effect on NK cell function is shown in Figure 1. 

To date, almost all viruses that have been found to infect NK cells are enveloped viruses, with the exception of TTV. This suggests that there is a bigger (evolutionary) advantage for enveloped viruses compared to naked viruses to infect NK cells and modulate anti-viral NK cell immunity. Cells infected with enveloped viruses display viral proteins on their membrane, making them prone to ADCC by NK cells [4]. Therefore, the inhibition of NK cell ADCC capacity may be specifically of importance as immune evasion strategy for enveloped viruses. 

It is apparent that viruses that cause systemic infections will encounter NK cells during an infection. However, infections with viruses like influenza and RSV are restricted to the lungs. Consistent with their important immunological role NK cells are distributed throughout the whole body, including the lungs [59]. Moreover, infection with influenza and RSV has been shown to lead to a pulmonary influx of NK cells in vivo [60,61,62,63]. This co-localization of NK cells and viruses during infection supports the potential benefit for viruses to evade the anti-viral NK cells response.

Generally, only a proportion of NK cells is infected in in vitro experiments. For viruses that enter NK cells through target cell interactions, the proximity of NK cells with infected cells seem to be the main determinant for infection. In the case of receptor-mediated entry, limited expression of specific entry receptors may result in non-homogeneous infection of NK cells, although other factors or phenotypic alterations may also be responsible. As NK cells are among the first cells to react to viral infections, inhibition or skewing of a small subset may already have a significant impact on the subsequent immune response. This is supported by increasing evidence showing that NK cells have an important role in shaping the adaptive immune response to viral infections (as reviewed in [64,65]). This also has implications for vaccine development, as attenuated viruses used for vaccination may not infect NK cells and therefore induce different skewing of the adaptive (memory) immune response compared to natural infection. With the limited data that is available it is impossible to determine whether this altered NK cell response would be beneficial or detrimental for protection.

A caveat of multiple studies that investigate NK cell infection is the lack of a clear distinction between the effect of infection as opposed to mere interaction. Incubation of NK cells with viral particles can lead to modulation of NK cell function in absence of infection. The mere interaction between viral particle and NK cell can be enough to trigger or inhibit certain effects. Whether infection is a prerequisite for NK cell modulation, can be studied using UV-inactivated viruses, as has been done for VV [43], or in the presence of a fusion inhibitor, as has been done for RSV [42]. However, in most of the abovementioned studies these controls were absent.

Several questions remain unresolved and need further investigation. The contribution of infected NK cells to disease severity and viral load remains unclear. Answering this question is challenging, as isolation of (local) cell subsets from patients is often impossible, making it difficult to demonstrate infected NK cells in vivo. The potential use of animal models is limited, as NK cell receptor repertoires differ between species. Moreover, many viruses are host-specific and infection in animals may not mimic the human situation. 

It is essential to expand our knowledge on possible cellular targets and reservoirs of viral infections. Intervention strategies to treat viral infections, such as viral entry inhibitors, may have different effects on particular cell types, as was demonstrated for influenza in murine NK cells [16]. Hence, the development of specific therapeutics could benefit from studies into viral infection of different cell types. In addition, viruses that are known to spread through cell–cell interactions may require alternative targets for drug development. The fact that viruses can infect NK cells suggests that this feature contributes to establishing a successful infection. Therapeutic inhibition of NK cell infection may therefore counteract the evasion strategies of the virus, potentially leading to an enhanced antiviral immune response. 

## Figures and Tables

**Table 1:** Overview of viruses known to infect natural killer (NK) cells.

Virus	Family	Entry Mechanism	Modulation of NK Cells	Productive Infection	Reference
Torque teno virus (TTV)	Anelloviridae	Unknown	Unknown	Yes	[31]
Human pegivirus (HPgV)	Flaviviridae	Unknown	↑ Survival ↓ IFN-γ production	Unknown	[32]
Cytomegalovirus (CMV)	Herpesviridae	Unknown	Unknown	No	[33]
Epstein Barr virus (EBV)	Herpesviridae	Acquisition of receptor after cell–cell interaction Receptor: CD21 Co-receptor: MHC-II	Morphological changes Transformation NK cell malignancies	No	[24,25,29,30,34,35]
Herpes simplex virus (HSV)	Herpesviridae	Cell-cell interaction with HSV-infected fibroblasts	Unknown	Unknown	[36]
Human herpesvirus 6 (HHV-6)	Herpesviridae	Unknown	↑ CD4 expression	Unknown	[23]
Varicella zoster virus (VZV)	Herpesviridae	Cell-cell interaction with VZV-infected epithelial cells	↑ CD57 expression ↑ Chemokine receptors ↓ CD56 expression ↓ FcγRIII expression	Yes	[37]
Influenza A virus (IAV)	Orthomyxoviridae	Clathrin- and caveolin-dependent endocytosis Receptor: sialic acids	↑ Apoptosis ↓ Cytotoxicity ↓ Cytotoxicity receptors ↓ Cytokines and chemokines	No	[14,15,18,38,39]
Measles virus (MV)	Paramyxoviridae	Unknown	↓ Cytotoxicity	Unknown	[40]
Respiratory syncytial virus (RSV)	Pneumoviridae	Possibly macropinocytosis Receptor: FcγRIIIA (RSV-antibody complexes)	↑ IFN-γ production ↑ KIR expression ↓ Cytotoxicity ↓Cytotoxicity receptors	No	[41,42]
Vaccinia virus (VV)	Poxviridae	Cell-cell interaction	↑ KIR signaling ↓ Cytotoxicity	No	[43,44]
Vesicular stomatitis virus (VSV)	Rhabdoviridae	Unknown	Unknown	Yes	[45]
Human immunodeficiency virus 1 (HIV-1)	Retroviridae	Receptor-mediated entry Receptor: CD4 Co-receptors: CXC4/CCR5	↑ Apoptosis	Yes	[19,20,21,22,46]
Human T-lymphotropic virus (HTLV)	Retroviridae	Cell-cell interaction with T cells	↑ Proliferation ↑ Survival	Unknown	[47,48,49]

FcγR, Fc gamma receptor; IFN, interferon; KIR, killer cell immunoglobulin-like receptor; MHC, major histocompatibility complex; ↑ increased; ↓ decreased.
